# Increasing Bike-Sharing Users’ Willingness to Pay — A Study of China Based on Perceived Value Theory and Structural Equation Model

**DOI:** 10.3389/fpsyg.2021.747462

**Published:** 2022-01-18

**Authors:** Hanning Song, Gaofeng Yin, Xihong Wan, Min Guo, Zhancai Xie, Jiafeng Gu

**Affiliations:** ^1^Institute of International Economy, University of International Business and Economics, Beijing, China; ^2^Dong Fureng Institute of Economic and Social Development, Wuhan University, Wuhan, China; ^3^School of Banking and Finance, University of International Business and Economics, Beijing, China; ^4^School of Marxism, Hebei University of Science and Technology, Shijiazhuang, China; ^5^School of Statistics, University of International Business and Economics, Beijing, China

**Keywords:** bike sharing, willingness to pay, perceived value, structural equation model, Likert-type scale

## Abstract

Bike sharing, as an innovative travel mode featured by mobile internet and sharing, offers a new transport mode for short trips and has a huge positive impact on urban transportation and environmental protection. However, bike-sharing operators face some operational challenges, especially in sustainable development and profitability. Studies show that the customers’ willingness to pay is a key factor affecting bike-sharing companies’ operating conditions. Based on the theories of perceived value, this study conducts an empirical analysis of factors that affect bike-sharing users’ willingness to pay for bike-sharing through measurement scales, user surveys, and structural equation models. We designed a five-point Likert-type scale containing 11 latent variables affecting willingness to pay and a total of 34 measurement items. We investigate bike-sharing users in China’s first and second-tier cities, with a total of 502 participants. The results show that perceived value, payment awareness, trust, and environmental awareness constitute key factors that directly affect bike-sharing users’ willingness to pay. And perceived usefulness, perceived ease-of-use, perceived cost, and perceived risk indirectly affect bike-sharing users’ willingness to pay. However, we found no significant effects of perceived entertainment on perceived value or word of mouth on willingness to pay. Our results are expected to provide theoretical and practical implications for bike-sharing programs.

## Introduction

Since 2014, a number of private, profit-making, app-based dockless bike sharing (hereafter referred to as bike sharing) has experienced a leap in growth within China (Chen Z. et al., 2020). These systems combine mobile payments and global positioning system (GPS) positioning and tracking technology. All necessary steps to use a bike, such as locating, unlocking a bike, recording a trip, and paying for the service come together into a mobile application (APP) (Shen et al., 2018). As a representative industry of the sharing economy, the bike-sharing industry advocates for the concept of “green environment protection” (Zhang and Mi, 2018). The bike-sharing industry also relies on information technology (IT) for bike management and expense payment (Fishman et al., 2015), which fully shows the characteristics of convenience, and carries the “last mile” of residents’ traffic (Xu et al., 2019; Nocera et al., 2021).

Quality bike-sharing systems can have a huge positive impact on urban transportation (Guidon et al., 2019). Currently, in many urban areas, the main challenge is to solve the problem of the widespread use of motor vehicles (Macioszek, 2019). Although bike-sharing systems are not specifically developed to lead people to change transport mode, shared bikes can be used on a quick and convenient basis, and a person’s decision about taking a trip can be made in a short time. Hence, shared bikes are suitable for short-distance trips (Zhao et al., 2015). In Poland and other countries that use shared bikes, bike-sharing systems are designed to encourage more people to commute using bicycles other than motor vehicles, thereby reducing congestion and enhancing transport accessibility (Macioszek and Kurek, 2020). In addition, bike-sharing systems are able to promote an alternative way of traveling and can be used as a way of enhancing sustainable urban mobility (Macioszek et al., 2020). That means bike-sharing benefits for less use of vehicles; better use of space for movement and parking; easing traffic congestion; cutting carbon emissions, reducing energy losses; extending the life of road networks; saving time and money for residents on short and medium distances; better access to urban services; enhancing the sustainable development of urban transportation (Drynda, 2014; Fishman et al., 2014).

However, with the rapid development of bike sharing in Chinese cities, some unsustainability issues have been exposed (Wang et al., 2019). First, the bike-sharing industry faces chaotic deposit management and large numbers of broken bikes (Guo et al., 2017). Second, piles of shared bikes scatter around the parking spaces increase the difficulty of regulation and distribution, directly lowering the quality of bike-sharing services. Due to the lack of regulation, many users park shared bikes at any place they like, leading to negative impacts such as blocking sidewalks, occupying common areas, and even encumbering other modes of transport (Chang et al., 2018; Shi et al., 2018). Third, regulations and supervision of the bike-sharing industry are imperfect, with large numbers of shared bikes vandalized or stolen. Fourth, no unified standards are available for the cost and quality of bike-sharing services, which means safety concerns such as traffic accidents and the leakage of personal information often cause trouble for bike-sharing users. Fifth, currently, the profit from the bike-sharing industry is narrow, as bike-sharing operators find it difficult to make profits relying merely on users’ service fees (Yang et al., 2019b), and the thin profit is far from meeting the needs of early investment and later maintenance of operators (Yan and Zhuang, 2019). These issues above have seriously affected the sustainability of Chinese bike-sharing operators. Since 2017, less competitive Chinese bike-sharing operators such as Wukong, Xiaolan, Kuqi, and Xiaoming have closed down or transferred (Xu, 2020). Giants of the bike-sharing industry also suffered from failure. The former leaders in the bike-sharing industry, Mobike and Ofo, had around 95% of the Chinese bike-sharing market in 2017 (Li, 2017). However, in 2018, Mobike was acquired by a Chinese group-buying giant for consumer products and retail services named Meituan (Yang et al., 2019a; Kittilaksanawong and Liu, 2021), and Ofo’s bike-sharing business took heavy losses and finally came to an end (Bieliński and Ważna, 2018; Ravi and Arora, 2019).

For the sustainable development of bike-sharing operators, enhancing the profitability of the bike-sharing industry is indispensable (Tian et al., 2021). Increasing the profitability includes enriching the profit model, raising service charges, and expanding the market demand for shared bikes (Liu and Wang, 2019). However, it is difficult to effectively solve a single profit model in the bike-sharing industry. At present, bike-sharing operators face fierce competition, and other earning modes are not yet mature, with problems such as scarce profits and lack of long-term profitability (Liu and Wang, 2019). Measures to raise service charges seem to do little to increase the profits of bike-sharing operators. Because shared bikes have elastic demand, users’ demand is likely to decrease significantly when service charge decreases. Once the service charges increase, users’ demand for shared bikes will reduce significantly, offsetting the positive effect of service charges increase on profitability. Compared with enhancing the profit model and raising service charges, expanding the market demand for shared bikes is a more effective way to boost the profits of bike-sharing operators. In 2019, the cyclist scale of Chinese bike-sharing services is about 259 million, and the market demand is relatively strong. In addition, the market scales of Chinese bike-sharing continued to grow steadily from 2016 to 2020 and there is still room for improvement in the demand of bike-sharing users (Xiao and Wang, 2020). To turn the strong market demand into operators’ profits, the key to the problem lies in improving the users’ willingness to pay for bike sharing. The higher the user’s willingness to pay for the service, the more likely the user is to choose a shared bike when traveling. Therefore, the study of bike-sharing users’ willingness to pay and its influencing factors is of great significance to solve the profit problem of bike-sharing operators.

In terms of the impact of users’ payment intention on bike-sharing operators, many studies have found that users’ willingness to pay affects the operation and management quality of bike sharing and strongly impacts the profitability of bike-sharing operators (Kim et al., 2017). Bike-sharing operators mainly depend on deposits, advertising, and user rent fees to make profits (Tian et al., 2021). Bike-sharing operators ever made profits from investment activities mainly through deposits paid by customers. However, some bike-sharing operators struggled to refund deposits to their users, which resulted in serious social problems (Sago, 2020). However, in 2019, the Ministry of Transport of China no longer allowed bike-sharing operators to collect user deposits “in principle” (Lo, 2019). In addition, since 2017, Beijing, Shanghai, Shenzhen, and other cities began to ban advertisements on shared bikes, and bike-sharing operators can no longer gain revenue through advertisements (Yue, 2017). As a result, the user rent fees have become the primary profit source for bike-sharing operators in the bike-sharing system, and users’ payment intention has become a key factor affecting the operating conditions of bike-sharing (Sheng et al., 2019). Since 2019, when many of the surviving bike-sharing operators increased user rent fees based upon the length of time they use shared bikes, maintaining users’ willingness to pay has become more and more important for operators to sustain profits (Sago, 2020).

In terms of the influencing factors of users’ willingness to pay, the customer is widely considered to be a critical direct influencing factor, significantly affecting users’ satisfaction and payment intention (Omar et al., 2011; Morar, 2013; Demirgünescedil, 2015). Perceived value is a user’s psychological assessment of a product or service after measuring their perception, which is usually affected by benefits and costs (Zeithaml, 1988). While enjoying a specific product or service, users can form a cognition of the benefits and costs and then estimate the total utility. Perceived value is a trade-off between the received benefits and sacrifices (Dodds et al., 1991; McDougall and Levesque, 2000). Perceived benefits are beliefs about positive consequences brought by a specific product or service, and perceived sacrifices refer to what is given up to enjoy a product or service (Fishbein and Ajzen, 1977; McMillen and Fisher, 1998). Studies indicate that perceived benefits generally include feelings of usefulness, ease-of-use, and entertainment (Davis, 1989; Blanco et al., 2010; Jiang et al., 2015; Komlan et al., 2016), while perceived sacrifices include the risk and all kinds of costs such as money, time, effort, and energy (Bauer, 1960; Morewedge et al., 2007; Benazić et al., 2015; Yang, 2017). These elements of perceived benefits and perceived benefits can be indirect factors affecting users’ willingness to pay (Chen, 2016; Lou et al., 2021). Boksberger and Melsen (2011) and Zhang and Deng (2018) pointed out that the key to measuring perceived value lies in the ratio between perceived benefits and perceived sacrifices. Besides perceived value, previous studies on bike-sharing also consider perceived trust, individual paying consciousness, word-of-mouth, and environment protection as direct influencing factors of bike-sharing users’ payment intention (Alford and Biswas, 2002; Ma et al., 2018; Cerutti et al., 2019; Xiao and Wang, 2020).

Based on the arguments above, this study builds a structural equation model around the influencing factors of users’ willingness to pay in bike sharing. This paper contributes to the future study of theoretical and practical aspects. In the theoretical aspect, there are some existing studies on bike-sharing users’ willingness to pay. However, few of these studies establish a systematic impact path of bike-sharing users’ payment intention. Based on these existing studies, this study summarizes the influencing factors of bike-sharing users’ payment intention and further analyzes the influencing factors of users’ perceived value — one key factor influencing users’ willingness to pay. Furthermore, we illustrate the formation mechanisms through building the impact path of bike-sharing users’ willingness to pay and perceived value. In practice aspect, it is expected that this study will help understand bike-sharing user consumption decision-making mechanisms by analyzing the impact path of users’ willingness to pay. Moreover, policy proposals on bike-sharing services can also be put up according to the impact path, which will facilitate increasing profits and promoting the sustainable development of bike-sharing operators.

## Overview of the Research

### Research Hypotheses

Willingness to pay is the maximum price or the possibility a customer is willing to pay in exchange for a product or service, which is an inner activity used to measure consumers’ subjective purchase intention for a particular commodity (Knetsch and Sinden, 1984; Varian, 1992; Wertenbroch and Skiera, 2002). As we discussed above, willingness to pay is a crucial factor affecting bike-sharing users’ consumptive decisions, and perceived value (i.e., perceived benefit and perceived sacrifice) significantly affects users’ willingness to pay. A bike-sharing user’s perceived value is the monetary and psychological evaluation of the utility, considering the gain and the pain of acquiring bike-sharing services (Kim et al., 2007). This article treats the attributes of usefulness, ease-of-use, and entertainment as perceived benefits of bike-sharing users’ perceived value, and treats perceived cost and perceived risk as perceived sacrifices. Furthermore, we introduce individual paying consciousness, word-of-mouth, perceived trust, and environmental protection as critical factors directly affecting users’ willingness to pay. Therefore, we put forward ten related hypotheses, as follows.

#### Perceived Value and Willingness to Pay

Studies clarified the positive impact of perceived value on users’ usage and payment intention toward bike-sharing services (Ma et al., 2018; Wu and Kim, 2019). Users’ enthusiasm for mobile payment, which is the primary consumption mode of bike sharing, can be influenced by adjusting perceived value (Yang et al., 2011; Ye et al., 2014). Some studies analyzed the cases and data of Chinese consumers’ mobile payment and agreed that perceived value is the overall utility evaluation after weighing income and paying, and consumers’ perceived value has strong links to payment intention (Zhong and Zhang, 2013; Zhao and Zhou, 2017; Zhao et al., 2018). Similar conclusions can be found in some studies on the transport service industry: the more favorable perception of the value of a transport service results in customers’ satisfaction and behavioral intention to accept the service, including bike sharing (Lai and Chen, 2011; Wang et al., 2018; Chen et al., 2021). Studies from Guangzhou, China revealed that individual psychological factors, including perceived value, imposed significant effects on bike-sharing users’ willingness to use and pay for shared bikes (Wei Z. et al., 2018; Gao et al., 2019). The stronger the users’ perceived value of a service or product, the higher the users’ willingness to pay for bike-sharing services. Hence, we propose the following hypothesis:

*H*_1_: Perceived value significantly positively influences the user’s willingness to pay.

#### Perceived Usefulness and Perceived Value

Perceived usefulness is the extent to which the behavior of enjoying bike-sharing services will be achieved efficiently (Van der Heijden, 2004; Venkatesh et al., 2012). Perceived usefulness is a key factor in adding bike-sharing service value, as shared bikes can be used to quickly and conveniently reach the destination in areas with little traffic (Li X. et al., 2018). Kuo and Yen (2009), Kim et al. (2010) and Slade et al. (2013) found that mobile payment users’ perceived value has a positive impact on their satisfaction and consumption willingness. Cheng et al. (2019) also argued the critical role of perceived usefulness in enhancing users’ attitudes toward bike-sharing programs. Therefore, this study expects that privacy risk has a negative effect on the intention to use it continuously. Therefore, this study expects that when paying for the use of shared bikes, if users perceive that bike sharing is useful, their perceived value will be higher.

*H*_2_: Perceived usefulness significantly positively influences users’ perceived value.

#### Perceived Entertainment and Perceived Value

Perceived entertainment in bike sharing is defined as the pleasure and relaxation enjoyed by users while using bike-sharing services, not just solving travel difficulties (Venkatesh et al., 2012). To expand new users and keep regular users operators launch creative activities to grow entertainment, such as “Free Riding Day” activities, which enrich users’ consumption experience and add entertainment value (Cao et al., 2018). According to the intrinsic motivation theory, perceived entertainment is considered as an internal motive for evoking a behavior (Davis et al., 1992; Lee et al., 2005). Roostika (2012) found that college students’ perceived value of mobile Internet positively correlates with their entertainment level. In the same way, if bike-sharing users perceive that they can obtain a higher level of riding pleasure or welfare by using bicycles, their perceived value may be higher (Zhang et al., 2017). Delightful experiences in bike-sharing services are critical for bike-sharing users to establish trust, satisfaction, and value for bike-sharing operators.

*H*_3_: Perceived entertainment significantly positively influences perceived value.

#### Perceived Ease-of-Use and Perceived Value

Perceived ease-of-use refers to the operation convenience of bike sharing (Van der Heijden, 2004). Specifically, this includes but is not limited to ease of consumption and ease of using shared bikes. For ease of consumption, users only download the APP before using it, and they can unlock the bike by scanning the QR code (Yin et al., 2019). Bike-sharing operators also cooperate with payment platforms such as WeChat and Alipay to help bike-sharing users complete the payments, thus simplifying the process of using shared bikes and enhancing the consumer experience. Such convenience makes people form positive attitudes to bike sharing, and take it as a life-benefiting tool for travel, which means higher perceived value. The easier bike-sharing service use is perceived, the more value they generate for bike-sharing users (Kim et al., 2009). Thus, the following hypothesis is developed:

*H*_4_: Perceived ease of use significantly positively influences perceived value.

#### Perceived Cost and Perceived Value

A bike-sharing user’s perceived cost is defined as the monetary cost which the user thinks he/she incurs by accepting bike-sharing services (Benazić et al., 2015; Huang et al., 2020). The monetary cost in bike-sharing services generally refers to the usage cost of bike sharing and the deposit (Sun and Duan, 2021). Studies suggest that consumers’ perceived cost has a reverse effect on their perceived value (Li, 2018; Li W. et al., 2018). Ma et al. (2020) suggest that “cheaper than other transport modes” is the primary reason for bike-sharing users to choose shared bikes, which means the low perceived cost is an essential factor to attract users to enjoy bike-sharing services (Ricci, 2015). Most bike-sharing operators no longer collect user deposits because the deposit fee is far more expensive than the service fee. Thus user deposits will substantially drive up users’ perceived cost, increasing the financial concern of users negatively impacting the perceived value of bike-sharing services (Zhao et al., 2020). Based on the discussion above, we argue that if bike-sharing users’ perceived cost is higher, their perceived value will decrease.

*H*_5_: Perceived value is significantly negatively affected by perceived cost.

#### Perceived Risk and Perceived Value

To a bike-sharing user, perceived risk is a feeling of uncertainty while enjoying bike-sharing services (Choi and Choi, 2020). Perceived risk refers to the perceived loss resulting from uncertainty (Wei Y. et al., 2018). A bike-sharing user’s perceived loss refers to the risk of defective bikes, traffic accidents, payment security, the leakage of personal information, etc. (Jacoby and Kaplan, 1972). In addition, a user’s perceived risk can also result from negative comments from other users and the media (Sun, 2018). If a user worries about the potential risk while using a shared bike, the user will be reluctant to believe the shared bike is valuable and reuse it. Thus, we propose the following hypothesis:

*H*_6_: Perceived value is significantly negatively affected by perceived risk.

#### Individual Paying Consciousness and Willingness to Pay

Individual paying consciousness refers to a customer’s willingness to pay a higher price for a more valuable product and service (Shirai, 2015; Rihn et al., 2018). Individual paying consciousness is negatively affected by price consciousness, which is defined as “the degree to which the consumer focuses exclusively on paying a low price” (Lichtenstein et al., 1993). A customer with low paying consciousness or high price consciousness would not like to pay a high price to a specific good or service, even though the good or service is valuable for him or her. That is because a customer with low paying consciousness can perceive emotional value and entertainment from low prices (Alford and Biswas, 2002). Prior studies and findings indicated that price promotion and discounts will result in lower-paying consciousness (Compeau and Grewal, 1998; Biswas et al., 1999). In China, at the beginning of the bike-sharing industry’s rise, bike-sharing operators promoted all kinds of incentives such as free riding and discounts to seize market share. As a result, bike-sharing users formed low-paying consciousness (i.e., high price consciousness), weakening their motivation to pay a reasonable price for bike-sharing services. And to make matters worse, a customer who lacks individual paying consciousness is prone to be a free rider (Baumol, 2004). As the number of free riders grows, it is not uncommon for them to steal and even destroy shared bikes (Berger, 2018). Nevertheless, if bike-sharing users have high individual paying consciousness, they will not mind paying a higher price for valuable services. As most bike-sharing operators in China have raised service fees since 2019, users with higher individual paying consciousness may have a stronger willingness to pay for bike-sharing services. Hence, the following hypothesis is proposed:

*H*_7_: Bike-sharing users’ individual paying consciousness significantly positively influences their willingness to pay.

#### Word-of-Mouth and Willingness to Pay

In bike-sharing services, word-of-mouth is the passing of information from one bike-sharing user to another. Whether the information is positive or negative largely depends on bike-sharing users’ satisfaction with the quality of bike-sharing service. Nongnuch (2014) pointed out in the exploration of user intention and relationship marketing that word-of-mouth plays a positive role in purchase desire and perceived value. For online shopping consumers, a study by Parry and Kawakami (2015) shows virtual word-of-mouth (the passing of information from person to person online) is positively correlated with perceived utilitarian value. Online consumer comments and ratings, which represent a common form of virtual word-of-mouth, have a direct positive effect on consumers’ willingness to pay (Bansal and Voyer, 2000; Nieto-García et al., 2017). Hence, we propose the eighth hypothesis:

*H*_8_: Word-of-mouth significantly positively influences bike-sharing users’ willingness to pay.

#### Perceived Trust and Willingness to Pay

A bike-sharing user’s perceived trust refers to the level of trust the user has in the quality of shared bikes (including comfort, safety, fast transport time, etc.), bike-sharing mobile APP user privacy protection, and the reliability of bike-sharing operators (Yuliati et al., 2020). Perceived trust significantly impacts users’ willingness to pay (Sirdeshmukh et al., 2002). Goudarzi et al. (2013) indicated that a customer’s trust positively affects their willingness to accept a certain service. Studies of online customers (including mobile APP users) come to the same conclusion: trust from online customers plays a positive role in promoting customers’ purchase and payment behavior (Alsajjan and Dennis, 2010; Simanjuntak et al., 2020). In bike-sharing services, a study from Indonesia argued that perceived trust significantly positively influences service usage and payment behavior (Yuliati et al., 2020). Therefore, we assume that the feeling of trust will make bike-sharing users predisposed to use and pay for shared bikes.

*H*_9_: Users’ perceived trust significantly positively influences their willingness to pay.

#### Environment Protection and Willingness to Pay

Cycling is considered to be an efficient way to reduce traffic congestion, noise, and air pollution, and promote environmental sustainability (Cupples and Ridley, 2008). Studies demonstrated that people with environmental consciousness tend to choose a more environmentally friendly transport mode, such as bike and public transport (Hunecke et al., 2001; Nordlund and Garvill, 2003; Shena et al., 2008; Kim et al., 2013). In the case of bike-sharing, we define environment protection as a bike-sharing user’s feeling of contributing to green transport and environment protection through the use of shared bikes. Kim et al. (2017) found that if people are aware of the positive effect of cycling to protect the ecological environment, it is possible for people to increase the use of bikes. It is assumed, therefore, that bike-sharing users will be more willing to use and pay for shared bikes if they have strong environmental consciousness.

*H*_10_: Users’ environmental awareness significantly positively affects their willingness to pay.

**FIGURE 1:**
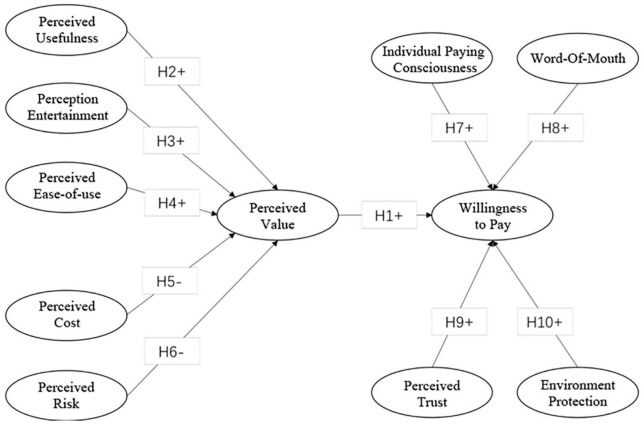
Theoretical framework.

Based on the above theoretical basis and hypotheses, we construct a theoretical framework presented in Figure 1.

### Materials and Methods

#### Scale Design

A scale is required to test whether the 10 hypotheses in the influencing factor model of users’ willingness to pay in bike-sharing are valid (Ma et al., 2019). The 10 hypotheses involve 11 latent variables, including perceived value (PV), perceived usefulness (PU), perceived entertainment (PE), perceived ease-of-use (PEU), perceived cost (PC), perceived risk (PR), individual paying consciousness (IPC), word-of-mouth (WOM), perceived trust (PT), environment protection (EP), and willingness to pay (WTP). The 11 latent variables are subdivided into 34 measurable variables, which correspond with 34 survey questions (see Table 1). All variables and survey questions were measured by a five-point Likert-type scale, which has been adopted in many behavioral intention studies (Chen and Chao, 2011; Cheng and Huang, 2013).

**TABLE 1:** Statistics of the measurement scale.

Latent Variable	Measured Variable	Item Description	References
Perceived Value (PV)	PV1	Compared with the money paid, it is worthwhile to pay for a shared bike	
	PV2	Compared with the physical strength, it is worthwhile to pay for a shared bike	Sirdeshmukh et al. (2002)
	PV3	Compared with the time spent, it is worthwhile to pay for a shared bike	
	PV4	In general, paying for a shared bike is valuable	

Perceived Usefulness (PU)	PU1	Bike sharing can improve travel efficiency	Slade et al. (2013), Liu and Tang (2015)
	PU2	I think bike sharing is useful	
	PU3	I think it is necessary to use a shared bike	

Perceived Entertainment (PE)	PE1	Bike sharing pleases me in riding	
	PE2	Bike sharing makes my life more interesting	Roostika (2012)
	PE3	Bike sharing is interesting, not boring	

Perceived ease-of-use (PEU)	PEU1	It is easy for me to use a shared bike	Slade et al. (2013), Liu and Tang (2015)
	PEU2	It doesn’t take much effort to learn to use a shared bike	
	PEU3	The paid use steps of the shared order are simple to operate	

Perceived Cost (PC)	PC1	Bike sharing’s pricing is relatively high	
	PC2	Excessive price is an obstacle to my use of a shared bike	Self-compiled
	PC3	I need to spend more money on traveling in bike sharing	

Perceived Risk (PR)	PC1	I will worry about the loss caused by the failure to refund the deposit in time	
	PC2	I’m worried that my safety will be damaged if I break down while riding a shared bike	Thakur and Srivastava (2014)
	PC3	I am worried that the company will collect my data without the customer’s permission and use it illegally	

Individual Paying Consciousness (IPC)	IPC1	I think paying fees can help me get higher quality service	
	IPC2	It is cost-effective to pay for valuable services	Self-compiled
	IPC3	I am willing to pay for services that I think are valuable	

Word-Of-Mouth (WOM)	WOM1	Before deciding to use the shared bike, I will watch the comments from the Internet and friends	Self-compiled
	WOM2	I prefer to spend money to ride a shared bike with better evaluation	
	WOM3	Comments from the Internet or friends around me will have an impact on my spending money to ride the brand’s shared bike	

Perceived Trust (PT)	PT1	I believe that the bike-sharing company I use can safely protect my funds (deposit)	
	PT2	I believe the quality of the shared bike I use is guaranteed	Self-compiled
	PT3	I believe that the company I use in bike sharing can do a good job in supporting the relevant services of the bike	

Environment Protection (EP)	EP1	I think bike sharing helps to travel green, save energy and reduce emissions	
	EP2	I think bike sharing can reduce the use of private cars or taxis	Self-compiled
	EP3	I am willing to pay for the use of bike sharing for environment protection	

Willingness to Pay (WTP)	WTP1	In the future, I may try (or continue) to pay for the use of bike sharing, such as buying bicycle monthly cards and annual cards, etc.	
	WTP2	If bike sharing is what I need to use, I am willing to pay for it	Fang et al. (2018)
	WTP3	I am willing to recommend high-quality shared bikes to my friends	

#### Data Collection and Variable Measurement

The scope of this survey is limited to the first-tier and second-tier cities in mainland China with high utilization rates in bike-sharing. We first conducted a pre-survey to prevent the distortion of the data collected by a large-scale survey. Because the overall preliminary test results in the pre-survey are ideal, we retained and included all the measurement items and survey results in the formal survey samples. In this paper, we collected a total of 502 questionnaires in the pre-investigation and investigation stages. Next, we cleaned the original data obtained from the questionnaires and eliminated invalid data to ensure the quality of the analysis samples. Finally, we got 458 available samples, accounting for 91.2% of the total. See Table 2 for the information of valid respondents.

**TABLE 2:** Descriptive statistics of sample data.

Statistical Items	Category	Frequency	Percentage (%)
Gender	Male	213	46.51

	Female	245	53.49
Age	19–25 years old	52	11.35
	26–30 years old	234	51.09
	31–40 years old	140	30.57
	40–50 years old	27	5.90
	Over 50 years old	5	1.09

Degree of education	Junior high school and below	5	1.09
	Senior high school	9	1.97
	College/Undergraduate	363	79.26
	Graduate student and above	81	17.69

Monthly usage frequency	0 times	56	12.23
	1–5 times	205	44.76
	6–10 times	93	20.31
	10–20 times	71	15.50
	More than 20 times	33	7.21

Duration of each use	Within five minutes	55	12.01
	5–10 min	201	43.89
	10–15 min	134	29.26
	15–30 min	57	12.45
	More than 30 min	11	2.40

According to the information of valid interviewees, there are a few more female users among interviewees, most of whom are under 30 years old. Less than 30% have a junior college or undergraduate degree, with the vast majority completing higher education. Users consume bike-sharing services once to five times a month (accounting for nearly 50%), indicating that most users use shared bikes as an unconventional means of transportation; the frequency of use is generally not high. In addition, respondents used bikes for five to ten minutes at a time (accounting for 43.9%). Thus, the survey group conforms to the general situation of users in bike-sharing.

## Empirical Test and Results

### Reliability Analysis

The reliability and validity of data samples are analyzed by IBM SPSS Statistics (New York, NY, United States). Reliability evaluates the accuracy and trustworthiness of experimental data. Before using a structural equation to analyze the model, it is necessary to examine the reliability of data samples to measure the model’s adaptability and effectiveness of hypothesis testing. The Cronbach’s α coefficient is selected as the measuring index of reliability (Lao and Wu, 2013). When Cronbach’s α coefficient is greater than 0.7 and less than 0.8, the scale’s reliability can be considered good (Xie and Han, 2005; Li and Tian, 2017). As shown in Table 3, Cronbach’s α coefficient and corrected item-total correlation (CITC)-value both fall into the category of high reliability. Furthermore, the coefficient does not increase significantly after removing items, indicating that the full scale has good reliability.

**TABLE 3:** Reliability analysis results.

Latent Variable	Measured Variable	Cronbach’s α	Corrected item-total correlation (CITC)	Cronbach’s α if Item Deleted
Perceived Value (PV)	PV1		0.864	0.888
	PV2		0.831	0.895
		0.922		
	PV3		0.784	0.912
	PV4		0.815	0.901

Perceived Usefulness (PU)	PU1		0.680	0.810
	PU2	0.838	0.753	0.730
	PU3		0.688	0.789

Perceived Entertainment (PE)	PE1		0.732	0.811
	PE2	0.861	0.793	0.751
	PE3		0.690	0.847

Perceived Ease-of-use (PEU)	PEU1		0.693	0.743
	PEU2	0.824	0.699	0.740
	PEU3		0.660	0.783

Perceived Cost (PC)	PC1		0.500	0.725
	PC2	0.739	0.659	0.536
	PC3		0.545	0.680

Perceived Risk (PR)	PC1		0.645	0.805
	PC2	0.828	0.780	0.670
	PC3		0.640	0.808

Individual Paying Consciousness (IPC)	IPC1		0.591	0.714
	IPC2	0.774	0.599	0.71
	IPC3		0.642	0.663

Word-Of-Mouth (WOM)	WOM1		0.682	0.856
	WOM2	0.859	0.782	0.759
	WOM3		0.743	0.794

Perceived Trust (PT)	PT1		0.783	0.887
	PT2	0.905	0.843	0.835
	PT3		0.809	0.864

Environment Protection (EP)	EP1		0.794	0.859
	EP2	0.898	0.809	0.849
	EP3		0.800	0.854

Willingness to Pay (WTP)	WTP1		0.857	0.911
	WTP2	0.935	0.855	0.913
	WTP3		0.883	0.891

### Validity Analysis

We measured the questionnaire’s validity from two aspects: content and structure. Content validity measures the content practicality and logical rationality of variables and items. The questionnaire used in this study comprehensively integrated the structural relationship of variables from the previous research literature, including reasonable text expression and the writing logic of the questions. Therefore, the measure used in our research has high content validity (Malmgreen et al., 2009).

#### Structural Validity Analysis

Structural validity is the degree to which the items in the questionnaire adequately reflect the measured variables. In this paper, we used exploratory factor analysis (EFA) to measure structural validity (Watkins, 2018). First, we conducted the Kaiser–Meyer–Olkin (KMO) test (Hill, 2011), resulting in 0.868 > 0.5. Next, we found the Bartlett spherical test *P*-value significantly less than 0.01. These results are very significant, which shows that the measurement data collected in this questionnaire has a good concentration and meets the prerequisite for further factor analysis.

Then, we carried out an exploratory factor analysis. We extracted 11 factors by calculation, and we obtained the explanatory rates of variance by rotation as follows: 9.887, 7.674, 7.614, 7.160, 7.133, 6.907, 6.829, 6.793, 6.581, 6.300, and 5.962%. The total explanatory power reached 78.840%, indicating that these 11 factors can better explain the information contained in the questionnaire data.

After maximum variance rotation, the absolute value of factor load of each measurement item was higher than 0.7, and the commonality of corresponding items was higher than 0.4. These findings indicate a significant correlation between research items and factors, and these factors can effectively extract information, thus confirming that the scale has good structural validity.

#### Confirmatory Factor Analysis and Correction

Confirmatory factor analysis (CFA) can further investigate the fit between actual measurement data and the theoretical framework, which is implemented in AMOS software (Brown and Moore, 2012; Brown, 2015). Before adopting this method, the first step is to describe the model of covariant relation and measure and revise it to increase its degree of fit. After measuring the model, we carried out the CFA using AMOS software. The results show that all fit parameters meet the requirements except adjusted goodness of fit index, AGFI = 0.881. This result is not up to 0.9 or higher. Therefore, it is necessary to modify the model to optimize and improve the model’s fit.

**FIGURE 2:**
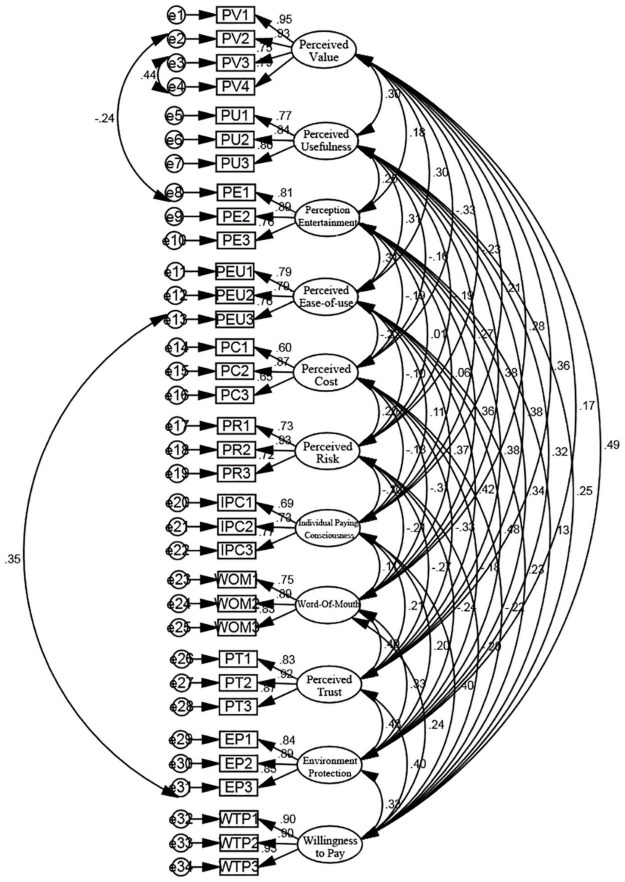
Modified measurement model of influencing factors of users’ willingness to pay in bike sharing.

The model needs to be re-estimated after three times of model modification to increase the correlation path. Figure 2 shows the modified model.

After conducting CFA on the modified model, the results of the modified model test were chi-square fit statistics/degree of freedom, CMIN/DF = 1.438 < 3, which meets the requirements. Therefore, comparative fit index (CFI), goodness of fit index (GFI), incremental fit index (IFI), tucker lewis index (TLI), normed fit index (NFI), and other values are greater than 0.9, which meets the standards. In addition, root mean square error of approximation (RMSEA) is 0.031 < 0.08, which shows a high degree of fit.

#### Analysis of Discriminant Validity and Convergent Validity

We provide an analysis of the discriminant and convergent validity of the internal items of each variable. The standardized factor load of confirmatory factor analysis on the modified model is higher than 0.5, and the significance level is high. The reliability of customers’ perceived usefulness, ease-of-use, entertainment, risk, cost, and value, as well as payment awareness, brand reputation, perceived trust, environment protection, and willingness to pay, are as follows: 0.845, 0.823, 0.862, 0.839, 0.753, 0.918, 0.777, 0.865, 0.905, 0.895, and 0.935, all of which are greater than 0.7. Average variance extracted (AVE) is 0.738, 0.646, 0.676, 0.608, 0.511, 0.639, 0.537, 0.682, 0.761, 0.741, and 0.828, respectively, all of which are greater than 0.6. These results meet the convergence effectiveness standard and have a high overall degree of fit, so it is possible to analyze all the variables next. See Table 4 for details. From the square root of AVE in the diagonal of the matrix (the bold value Table 4), the discriminant validity of the measuring scale is relatively good; that is, there are significant differences among potential variables.

**TABLE 4:** Correlation analysis and differential validity.

	1	2	3	4	5	6	7	8	9	10	11
Perceived Usefulness	**0.803**										
Perceived Entertainment	0.226**	**0.822**									
Perceived Ease-of-use	0.265**	0.262**	**0.779**								
Perceived Cost	−0.139**	−0.160**	−0.161**	**0.714**							
Perceived Risk	−0.199**	−0.007	−0.083	0.175**	**0.799**						
Individual Paying Consciousness	0.225**	0.039	0.047	−0.081	−0.093*	**0.732**					
Word-of-Mouth	.0352**	0.337**	0.325**	−0.255**	−0.200**	0.098*	**0.825**				
Perceived Trust	0.329**	0.336**	0.363**	−0.276**	−0.261**	0.183**	0.353**	**0.872**			
Environment Protection	0.273**	0.307**	0.426**	−0.142**	−0.207**	0.174**	0.295**	0.376**	**0.860**		
Perceived Value	0.303**	0.171**	0.263**	−0.292**	−0.245**	0.163**	0.247**	0.338**	0.157**	**0.859**	
Willingness to Pay	0.220**	0.114*	0.192**	−0.183**	−0.182**	0.347**	0.233**	0.373**	0.297**	0.452**	**0.909**

*1, Perceived Usefulness; 2, Perceived Entertainment; 3, Perceived Ease-of-use; 4, Perceived Cost; 5, Perceived Risk; 6, Individual Paying Consciousness; 7, Word-of-mouth; 8, Perceived Trust; 9, Environment Protection; 10, Perceived Value, 11 = Willingness to Pay.*

* suggests the significance at the level of 10%, ** suggests the significance at the level of 5%. The bold value means the square root of the average variance extracted (AVE).

The above test results show that the measurement model in this paper can effectively fit various influencing factors of users’ willingness to pay, with high reliability and validity. We can analyze the structural equation model based on the model and sample data to check the path relationship among potential variables and verify theoretical assumptions.

### Structural Equation Model

#### Model Building

**FIGURE 3:**
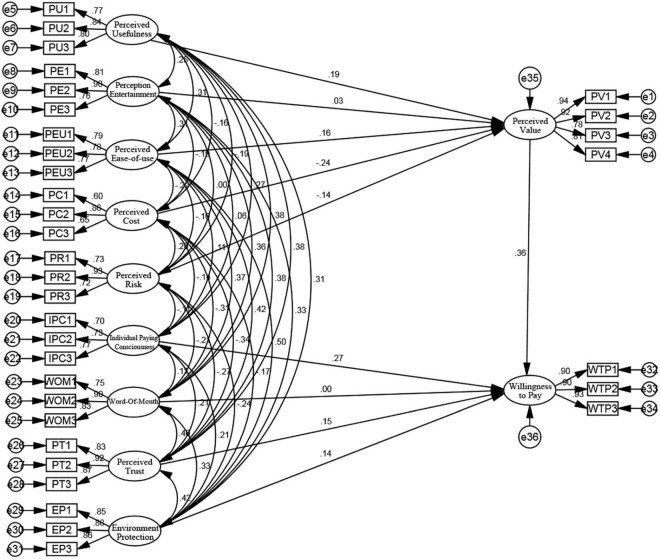
Structural equation analysis model.

The structural equation model, integrating factor analysis and path analysis, can be used for analyzing direct and indirect relations among variables (Kaplan et al., 2015). The structural equation includes two equations: the measurement equation that describes the relationship between the latent variable and measured variable, and the structural equation that describes the relationship between latent variables (Fishbein and Ajzen, 1977). This paper uses the structural equation model to build a hypothetical model about the influencing factors of bike-sharing users’ willingness to pay. We introduce AMOS to build the structural equation. Figure 3 shows the specific structural equation analysis model 3, consisting of 11 latent variables, 34 measurable variables, and 36 residual variables.

Our study uses AMOS to analyze the degree of fit, and the results show that CMIN/DF = 1.714 < 3, with the fit between the model and sample data reaching the standard. In addition, CFI, GFI, and other values also reached the standard because they were all higher than 0.9. RMSEA also meets the standard at 0.040 < 0.08. However, the AGFI-value is 0.880; it does not reach the standard above 0.9. Assuming that the model fitting degree does not reach the optimal standard, the model needs to be modified and optimized.

Looking at the path coefficient of the structural equation model, we get Table 5. We find that the *P*-value of two paths except for perceived entertainment→perceived value and word of mouth→willingness to pay is greater than the significant standard of 0.05. This finding indicates that perceived entertainment and word of mouth have no significant influence on willingness to pay at the significance level of 5%. Therefore, hypotheses *H*_3_ and *H*_8_ are not valid. *P*-values of other paths are less than 0.05 significant standard, indicating that they significantly influence at the 5% significance level. This finding supports the other hypotheses.

**TABLE 5:** Path coefficient of structural equation model.

Path relation			Standardized path coefficient	*T*-Value	*P*-Value	Hypothesis
Perceived Value	←	Perceived Usefulness	0.185	3.488	0.002	Supported
Perceived Value	←	Perceived Entertainment	0.033	0.655	0.513	Not Supported
Perceived Value	←	Perceived Ease-of-use	0.162	2.982	0.003	Supported
Perceived Value	←	Perceived Cost	−0.241	−4.436	0.001	Supported
Perceived Value	←	Perceived Risk	−0.135	−2.77	0.006	Supported
Willingness to Pay	←	Individual Paying Consciousness	0.27	5.442	0.001	Supported
Willingness to Pay	←	Word-of-Mouth	0.001	0.029	0.977	Not Supported
Willingness to Pay	←	Perceived Trust	0.155	3.027	0.002	Supported
Willingness to Pay	←	Environment Protection	0.138	2.814	0.005	Supported
Willingness to Pay	←	Perceived Value	0.364	8.152	0.000	Supported

**FIGURE 4:**
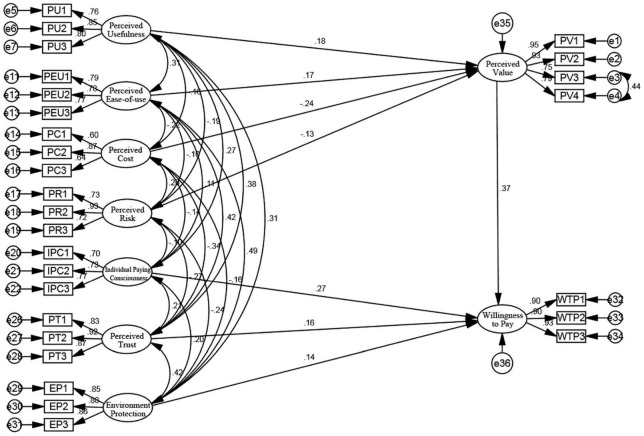
Modified structural equation analysis model.

Since perceived entertainment and word-of-mouth have no significant influence on willingness to pay at a significance level of 5%, thereby not supporting the hypothesis, it is necessary to delete the two paths of perceived entertainment→perceived value and word-of-mouth→willingness to pay as the initial revision of the model. After deletion, we execute the model again and analyze the fitting degree of the model. Except for AGFI = 0.89 < 0.9, which does not meet the requirements, the remaining variables are in the acceptable range, thereby optimizing the model again. At this time, the *P*-value of each path has reached the significant standard of less than 0.05, supporting the hypothesis corrected by the residual covariance method. In addition, we found that we could reduce the chi-square value by adding a path to improve the model between two residuals. The correlation between variables starts with the variable with the largest MI-value, and finally, adding the path between e3 and e4 to modify the model. After correcting the model, we executed it again to get Figure 4:

After analyzing the fitting degree of the model again, we found CMIN/DF = 1.566 < 3, which meets the standard. At the same time, CFI and GFI are all higher than 0.9, reaching the standard. In addition, RMSEA is 0.035 < 0.08, and the relevant indexes of fitting degrees are all up to the standard. Therefore, the revised model has a high degree of adaptation.

#### Path Analysis and Hypothesis Test

After the model fitting test, it is necessary to analyze the model path, calculate each potential variable’s significant coefficient and path coefficient, and examine whether the hypothesis passes the test. Finally, based on the findings, verify the relationship between potential variables proposed in this study. See Table 6 for the conclusion of the research hypotheses after path analysis.

**TABLE 6:** Hypothesis test results.

Path relation			Standardized path coefficient	*T*-Value	*P*-Value	Hypothesis
Perceived Value	←	Perceived Usefulness	0.181	3.463	0.001	Supported
Perceived Value	←	Perceived Ease-of-use	0.174	3.284	0.001	Supported
Perceived Value	←	Perceived Cost	−0.238	−4.426	0.000	Supported
Perceived Value	←	Perceived Risk	−0.126	−2.605	0.009	Supported
Willingness to Pay	←	Individual Paying Consciousness	0.268	5.402	0.000	Supported
Willingness to Pay	←	Perceived Trust	0.158	3.251	0.001	Supported
Willingness to Pay	←	Environment Protection	0.140	2.892	0.004	Supported
Willingness to Pay	←	Perceived Value	0.365	8.294	0.000	Supported

The *P*-values of *t* in Table 6 are all less than 0.05, which means all hypothesis test results reach statistical significance, indicating:

• Impact on perceived value:

o Perceived usefulness has a significant positive effect, assuming *H*_2_ holds.
o Perceived ease-of-use has a significant positive effect, assuming that *H*_4_ holds.
o Perceived cost has a significant negative effect, assuming *H*_5_ holds.
o Perceived risk has a significant negative effect, assuming *H*_6_ holds.

• Impact on willingness to pay:

o Payment awareness has a significant positive effect, assuming *H*_7_ holds.
o Perceived trust has a significant positive effect, assuming *H*_9_ holds.
o Environment protection has a significant positive effect, assuming that *H*_10_ holds.
o Perceived value has a significant positive effect, assuming *H*_1_ holds.

**FIGURE 5:**
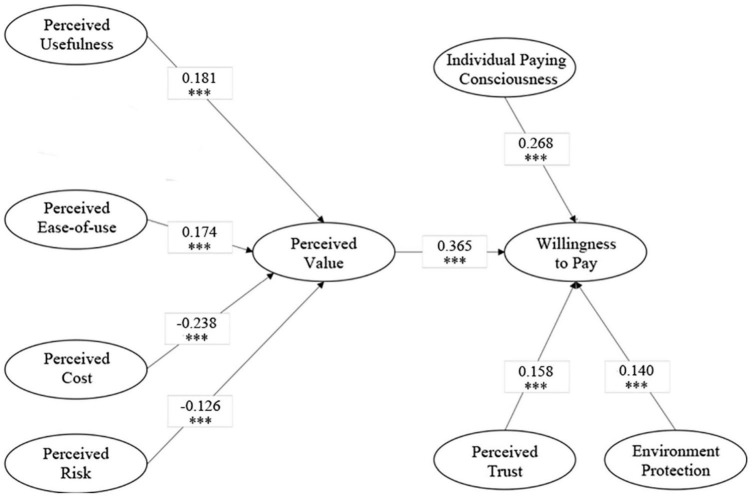
Influence path of modified structure equation model. ^***^ suggests the significance at the level of 1%.

At the same time, we can see that assuming *H*_3_ and *H*_8_ are not significant, that is, perceived entertainment has no significant impact on perceived value, and word-of-mouth has no significant effect on users’ willingness to pay. The influence path of the eight factors (perceived entertainment and word-of-mouth are excluded) is shown in Figure 5.

## Conclusion and Discussion

Based on the survey data of first-tier and second-tier cities in mainland China, perceived value theory, and structural equation model, this paper analyzed the direct and indirect factors that affect bike-sharing users’ willingness to pay. The results show that: first, perceived value, individual paying consciousness, perceived trust, and environmental protection have a significant and positive direct effect on users’ willingness to pay, with direct influence values of 0.365, 0.268, 0.158, and 0.140. While the positive effect of word-of-mouth on users’ willingness to pay is not significant. Second, perceived usefulness and perceived ease-of-use have significant and positive direct effects on perceived value (i.e., significant and positive indirect effects on users’ willingness to pay), with direct influence values of 0.181 and 0.174. Third, perceived cost and perceived risk have significant and negative direct effects on perceived value (i.e., significant and negative indirect effects on users’ willingness to pay), with direct influence values of −0.238 and −0.126. However, the impact of perceived entertainment on perceived value is not significant. Therefore, the main conclusions and proposes are given below:

### Factors Influencing Willingness to Pay

Perceived value is the crucial factor influencing bike-sharing users’ payment intention with the highest influence value of 0.365, and the conclusion is consistent with some studies on China’s bike-sharing programs such as Ma et al. (2018, 2019), and Ji et al. (2021). This means bike-sharing users put the utility gained from bike-sharing services first. Therefore, to attract more non-users and keep users, bike-sharing operators should focus more on users’ subjective well-being.

It is not difficult to understand why individual paying consciousness, with an influence value of 0.268, is the second most important factor to influence users’ payment intention. Many studies indicate that users’ perceived value significantly influences the usage behavior, however, it does not mean they are willing to pay a reasonable price for the valuable product or service they think. As we have discussed, sales promotion and discounts conducted by bike-sharing operators will make people seek lower prices and even become “free riders”, severely diminishing the willingness to pay. Bike-sharing operators should adhere to the bottom line to improve service quality and customer experience, refine the service and management, and not set off a “price war” to seize market share. Operators should also cultivate the individual payment consciousness on the whole market by improving users’ satisfaction.

Perceived trust, with an influence value of 0.158, can positively influence bike-sharing users’ willingness to pay. Bike-sharing users’ trust in the reliability and security of bike-sharing operators is a significant part of shaping a long-term relationship between users and operators (Gefen et al., 2003). Hence, we suggest that bike-sharing enterprises increase investment in human resources, capital, and technology. These investments will help maintain customer information, personal safety, and security of customer funds, improve the security of the service platform, ensure the related payment accounts of users, provide high-quality vehicles, and improve service reliability. To regulate bike-sharing operators’ behavior, it is also necessary for the government to enact laws governing the bike-sharing market (Ma et al., 2018).

Environment protection positively influences bike-sharing users’ willingness to pay with an influence coefficient of 0.140: The results of our study emphasize the importance of making people perceive the environmental value of bike sharing. To encourage cycling, some promotional campaigns aimed at promoting low-carbon and environment-friendly life are necessary (Geus et al., 2008). Some studies emphasized that bike-sharing operators’ marketing, news media’s presentation of bike-sharing, and some political ways can increase people’s environmental concern and create awareness about the positive aspects of using shared bikes (Brög et al., 2002; Berthoû, 2013; Zanotto, 2014). Therefore, we suggest that operators fully play the advantages of low-carbon environmental protection in bike sharing, strengthen the publicity and marketing of green travel elements in bike sharing, and carry out environmental protection activities. For instance, bike-sharing operators could organize “Earth Hour” in conjunction with green public welfare organizations and the public media to make bike-sharing users realize their contribution to environmental protection. Operators could also find ways to increase cooperation with successful public welfare and environment protection digital platform, such as Alipay “Ant Forest” (Chen B. et al., 2020; Yang et al., 2018).

### Factors Influencing Perceived Value

In terms of the four factors influencing users’ perceived value, the influence value of perceived cost is significantly higher than the other three factors. According to the prospect theory developed by Kahneman and Tversky (2013), people value gains and losses differently because of risk aversion. People have a tendency to avoid losses rather than gain benefits because the pain of perceived monetary loss (i.e., perceived cost) is greater than the satisfaction of an equivalent monetary gain. Therefore, decision-makers should concern more about the perceived cost to enhance bike-sharing users’ perceived value. It is difficult to cut bike-sharing users’ costs through reducing service fees because the low price strategy is no longer attractive to users and will significantly hurt operators’ profitability. In this context, it is suggested that bike-sharing operators engage in price discrimination, providing active users with huge discounts to stimulate more people to use shared bikes (Haider et al., 2018). For example, operators may consider introducing a membership upgrade system, in which the membership level goes up as the user use shared bikes more frequently. In addition, bike-sharing operators could carry out differentiated pricing at different membership levels or provide various preferential efforts. The benefit is that high-level users can enjoy lower prices and lower-level users will make greater preferential efforts, thus increasing their willingness to use and pay for shared bikes.

The other three factors, perceived usefulness, perceived ease-of-use, and perceived risk also have effects on bike-sharing users’ perceived value. Hence, we put out some policy proposals below.

Perceived usefulness first, we suggest that bike-sharing operators should devote themselves to optimizing basic services, optimizing the bike ownership rate and bike scheduling efficiency when users want to use bikes, and enhancing the practical value of bike sharing. Second, operators should publicize bike sharing’s advantages in losing weight and keeping fit, deepen users’ recognition of the usefulness of bike sharing, and then improve customers’ willingness to pay. Third, we suggest that the government better promote bike sharing by contrasting its advantages compared with other transport modes.

Perceived ease-of-use for one thing, bike-sharing operators should optimize the user experience, including improving client convenience, removing unnecessary functions, and avoiding cumbersome operations when users rent and return shared bikes. Bike-sharing operators should also focus on solving problems of huge piles of broken bicycles, limited service areas, and unreasonable divisions of parking places to improve bike-sharing users’ perceived ease of use while using shared bikes. For another, bike-sharing users’ behavior should also be supervised to enhance the convenience of using shared bikes. For example, some illegal behaviors, such as stealing and vandalizing shared bikes, should be integrated into the individual credit system *via* Internet. People whose illegal behaviors are recorded in the individual credit system will lose some credit scores. And they will not be able to enjoy bike-sharing services if their credit scores are lower than a certain degree, which can reduce bike-sharing uses’ illegal behaviors on shared bikes (Ji et al., 2021).

Perceived risk the influence value of perceived risks is relatively low, one possible reason is that the bike-sharing market and behavior of operators are becoming more regulated (Reddick et al., 2020). Nevertheless, some measures can still be taken to enhance users’ perceived value. For bike-sharing operators who still collect user deposits (Some electric bike sharing operators still collect deposits because of high operating costs), they should improve the measures for handling deposit risks and inform or unconditionally exempt customers. Operators could conduct scientific analysis in advance to protect users’ privacy and avert potential safety hazards and legal disputes during the registration and use of bike sharing. In addition, operators could improve the response mechanism for accidental injury and reflect it in the user service agreement, transforming it into an integral part of customer service. For example, to reduce the troubles and doubts caused by the risks that may occur during the use of bike sharing, operators can consider cooperating with insurance companies to provide paid cycling insurance to customers.

In the end, there are still some limitations in this study. Based on these limitations, some important future research priorities can be identified. On one hand, the questionnaire should cover more people and more places in China, especially for some small cities, counties, and even some rural areas in which bike-sharing services have become popular. On the other hand, this paper focused on bike-sharing users’ willingness to pay based on the structural equation model, and the impact of some realistic factors on users’ payment intention was still ignored. Future studies could further analyze these realistic factors. For instance, although there are very few dock bike-sharing programs, they may have an effect on users’ willingness to pay. Researchers could study the comparison between dockless and dock bike-sharing services to find out whether dock bike-sharing services will become a new competitive business model for China’s bike-sharing operators. Other realistic factors include discarded bike disposal, shared bike quantity control, legislation, support of the government, etc., which can be used to further analyze the impact on bike-sharing users’ willingness to pay.

## Data Availability Statement

The original contributions presented in the study are included in the article/supplementary material, further inquiries can be directed to the corresponding author/s.

## Ethics Statement

Ethical review and approval was not required for the study on human participants in accordance with the local legislation and institutional requirements. The patients/participants provided their written informed consent to participate in this study.

## Author Contributions

HS, GY, and JG conceived and designed the study. XW, MG, and ZX finalized this manuscript. HS and GY helped with data processing. JG participated in scale design, questionnaire distribution, and collection. All authors have read and approved the final manuscript.

## Conflict of Interest

The authors declare that the research was conducted in the absence of any commercial or financial relationships that could be construed as a potential conflict of interest.

## Publisher’s Note

All claims expressed in this article are solely those of the authors and do not necessarily represent those of their affiliated organizations, or those of the publisher, the editors and the reviewers. Any product that may be evaluated in this article, or claim that may be made by its manufacturer, is not guaranteed or endorsed by the publisher.
